# St. Louis Hospital Reports

**Published:** 1843-06

**Authors:** 


### ST. LOUIS HOSPITAL REPORTS.—ST. LOUIS MEDICAL AND SURGICAL JOURNAL.

When our first article went to press, we hoped and believed that it was but the first of a series from the pen of Dr. Linton. Since that time, however, we have received the first and second numbers of the *St. Louis Medical and Surgical Journal*, edited by Dr. Linton. Of course his pen will hereafter be devoted to the enriching of the columns of his own Journal. The latter is a monthly of sixteen octavo pages, good paper, fair type—our only regret at its appearance is its depriving us of an old and valued correspondent. C.

